# Early Detection and Assessment of Covid-19

**DOI:** 10.3389/fmed.2020.00311

**Published:** 2020-06-09

**Authors:** Hafiz Abdul Sattar Hashmi, Hafiz Muhammad Asif

**Affiliations:** University College of Conventional Medicine, Faculty of Pharmacy & Alternative Medicine, The Islamia University of Bahawalpur, Bahawalpur, Pakistan

**Keywords:** SARS-Cov-2, Hashmi-Asif Covid-19 Chart, incubation, leukopenia, lymphopenia, thrombopenia, morbidity

## Abstract

**Background:** Since the Covid-19 global pandemic emerged, developing countries have been facing multiple challenges over its diagnosis. We aimed to establish a relationship between the signs and symptoms of COVID-19 for early detection and assessment to reduce the transmission rate of SARS-Cov-2.

**Methods:** We collected published data on the clinical features of Covid-19 retrospectively and categorized them into physical and blood biomarkers. Common features were assigned scores by the Borg scoring method with slight modifications and were incorporated into a newly-developed Hashmi-Asif Covid-19 assessment Chart. Correlations between signs and symptoms with the development of Covid-19 was assessed by Pearson correlation and Spearman Correlation coefficient (rho). Linear regression analysis was employed to assess the highest correlating features. The frequency of signs and symptoms in developing Covid-19 was assessed through Chi-square test two tailed with Cramer's V strength. Changes in signs and symptoms were incorporated into a chart that consisted of four tiers representing disease stages.

**Results:** Data from 10,172 Covid-19 laboratory confirmed cases showed a correlation with Fever in 43.9% (*P* = 0.000) cases, cough 54.08% and dry mucus 25.68% equally significant (*P* = 0.000), Hyperemic pharyngeal mucus membrane 17.92% (*P* = 0.005), leukopenia 28.11% (*P* = 0.000), lymphopenia 64.35% (*P* = 0.000), thrombopenia 35.49% (*P* = 0.000), elevated Alanine aminotransferase 50.02% (*P* = 0.000), and Aspartate aminotransferase 34.49% (*P* = 0.000). The chart exhibited a maximum scoring of 39. Normal tier scoring was ≤ 12/39, mild state scoring was 13–22/39, and star values scoring was ≥7/15; this latter category on the chart means Covid-19 is progressing and quarantine should be adopted. Moderate stage scored 23–33 and severe scored 34–39 in the chart.

**Conclusion:** The Hashmi-Asif Covid-19 Chart is significant in assessing subclinical and clinical stages of Covid-19 to reduce the transmission rate.

## Background

More than 571,678 people have been infected by Covid-19 and the death toll has reached 26,494 as of March 28th 2020, with 62,514 new daily cases reported in 24 h and deaths of 3,159 worldwide (1). After the initial epidemic appeared in China, it spread to dozens of other countries. Coronavirus disease (Covid-19), which is caused by a novel pathogen Severe Acute Respiratory Syndrome Coronavirus 2 (SARS-Cov-2), caused the current global pandemic (2). During this pandemic, the most critical questions aroused pertains to patients and clinicians in understanding how the disease spread to cause an epidemic, what its clinical presentation with a severity profile is, what assessment or diagnostic measures should be used, and what projected treatments and influences to prognosis and recurrence there are.

Covid-19 has threatened the entire world. For the health services providers, it became a challenge to make rapid forward planning to evaluate the transmission rate of SARS-Cov-2 without ready access to diagnostic techniques and future planning based on the sustainability of healthcare systems to cope with the outbreak (3). Pragmatic understanding of the novel pathogen SARS-Cov-2 revealed an essential genetic sequencing similarity to the previously known pathogen, SARS (4). A mean incubation period of 5.2 days of SARS-Cov-2 has been reported to cause the onset of symptoms and a mean 12.5 days for hospitalization from day of infection (5, 6). Fauci et al. emphasized the time interval during the incubation of SARS-Cov-2 to hold crucial information on pathogenesis and asserted the need to understand it to design an effective containment policy (7). Current understanding of Covid-19 pathogenesis focuses on the Angiotensin Converting Enzyme 2(ACE2) based SARS-Cov-2 cell entry that infects lung epithelial cells and synergistic entry through endosome proteases cell prime entry that infects the host cell (8). Novel coronavirus also infects T-lymphocytes (9). Recent retrospective studies revealed that elders are more prone to Covid-19 and were more likely to require invasive mechanical ventilation with a high mortality among Covid-19 infected patients, and robust research revealed the clinical presentation of Covid-19. Currently, Covid-19 is detectable with Reverse Transcriptase Polymerase Chain Reaction (RT-PCR), which detects presence of genetic fragments of SARS-Cov-2 within secretions from nasal and pharyngeal epithelial mucus membrane. Employed techniques of RT-PCR and immunoglobulin presence detection methods have their own limitations of detection within a specific time period. Prior to detection through RT-PCR, no method is available to assess Covid-19 infection during incubation and after the onset of symptoms. Consequently, a high transmission rate has been reported and needs to be reduced for effective containment (7). In this study, we evaluated the current knowledge of Covid-19 pathogenesis and its manifestation to formulate an easy method to detect and assess the Covid-19 course of infection and to counter outbreaks by reducing transmission rates through early sensing and adopting appropriate measures.

Comparatively similar clinical features were previously reported to be caused by influenza. Influenza, caused by H1N1, H3N2, and H5N1, produced variable symptoms in humans. Median incubation periods are 2 days, 1–6 days, and 2–5 days, respectively. All strains cause acute symptoms variable in nature and intensity (10–16). H1N1 causes a fever similar to H3N2, with a relatively shorter duration of 1–2 days while H3N2 causes a fever of 1–6 days. Avian influenza (H5N1) presents with baffling symptoms aggressive in nature, like inexplicable diarrhea or encephalopathy. Intensity of the symptoms is high and related with areas of known outbreaks. Fever (temperature > 38°C) is present in symptomatic patients with abdominal features including vomiting, diarrhea, myalgia or arthralgia, rhinorrhea, cough, and sputum production. All signs and symptoms appeared concomitantly on median 2 or 3 days after infection (10). H1N1 causes symptoms to appear on day 2. The virus is detectable during a median period of 2–6 days after infection. Sore throat, nasal congestion, nausea, vomiting, and myalgia are common symptoms with a mild to severe fever. Distinguishing signs are enlarged lymph nodes, tonsillitis, and throat congestion while prominent features are leukopenia, lymphopenia, and hypokalemia (11, 12). H3N2 significantly reduced the weight of patients during the early days of infection (13, 14). Severe cases of H5N1 presents with cardiomyopathies, ventricular tachycardia, renal failure, ventilation assisted viral pneumonia, Reye's syndrome, and pneumothorax. Death occurred due to multi-organ failure. Blood biomarkers abruptly developed leukopenia, lymphopenia, thrombocytopenia, and elevated aminotransferases (15, 16).

Clinical manifestations of SARS-Cov-2 appeared variable as compared to influenza. Symptoms of Covid-19 also vary slightly from region to region. Abdominal symptoms were more frequent in the USA than China (17–21). Asymptomatic, mild, and severe symptoms were observed in various studies (22–27). Asymptomatic or milder cases did not seek medical intervention; mild symptoms included a temperature >37.5°C and dry cough initially and could develop to moderate symptomatic cases. Fever, cough, abdominal discomfort, and deranged blood biomarkers were recorded in moderate cases. Severe cases presented with shortness of breath, dyspnea, and tachypnea and required mechanical ventilation (28). Persistent cough, fever, and fatigue were associated symptoms of an underlying pathology or pre-existing pathology not restricted to cardiovascular issues, hypertension, liver compromise, and diabetes. Blood pO_2_ levels decreased. Blood biomarkers developed lymphopenia, thrombopenia, and elevated aminotransferases in moderate and severe cases. White blood cells deteriorated in severe cases and required mechanical ventilation. Persistent fever and characteristic consistent coughing—initially dry for several days followed by a productive cough—are the main features in patients with pre-existing respiratory infections; a few symptoms were variable with geographical regions (29–38). In the current study we emphasized the pathogenesis of Covid-19 assessed through signs and symptoms and its manifestation to formulate a practicable approach to detect and assess Covid-19's course of infection to counter outbreaks by reducing the transmission rate through early sensing and adopting appropriate measures.

## Methods

### Data Collection

We used a retrospective approach to collect observational data about the most common presenting signs and symptoms in reported cases of Covid-19. Data was searched with the terms “clinical presentation of Covid-19, Clinical features of Covid-19, Covid-19 reported cases, clinical picture of Covid-19, Covid-19 symptoms” through search engines like Google Scholar, Pubmed, and Science Direct to obtain any available updated information about the clinical aspects and clinical presentation of Covid-19.

### Interpretation of Data

Data was assessed for common presentations made by collected publications for sensing essential common symptoms. The Hashmi-Asif Covid-19 Formula was designed based on collected data that adhered to the most common and easily accessible symptoms which can affect an early diagnosis of Covid-19 or, due to their absence, could delay diagnosis or cause misdiagnosis. The important differentiating clinical features, signs, and symptoms were aligned in table form providing a sketch of the most common essential symptoms. Data about the frequency of symptoms in relation with Covid-19 diagnosis were categorized into clinical features and blood biomarkers. We categorized common symptoms and blood biomarkers for Covid-19 extracted from the collected data and these were categorized into two groups.

### Classification of Data

The classification of normal to severe symptoms was determined from collected data containing values, ratios with interquartile ranges, and percentages of occurrence in observational studies. Four scoring tiers were formulated. Each sign and symptom were assigned a score by using the Borg Scale scoring method previously described by Hommerding et al. (39) with slight modifications. Signs and symptoms were given a score between 1 and 4. Normal signs and symptoms were given a score of 1 and placed in the first tier, mild presentation in signs and symptoms were given a score of 2 and placed in the second tier, third tier includes moderately presenting symptoms given a score of 3, and severe cases were given a score of 4 in the fourth tier. The highest score in the fourth tier scores 39 which represents severe disease while the lowest in the first tier scores 11 and showed normal or no disease. Mild disease scored between 13 and 22 and moderate disease scored between 23 and 33. Variable scoring showed stages of the disease as mild, moderate, or severe. Minimum and maximum scores were calculated and evaluated for the available data collected and compiled in Table 1. All data were calculated on the score chart to evaluate its efficacy for detecting early common signs and symptoms to make an easy decision on whether to hold isolation and other immediate measures surrounding the early confirmation of Covid-19. The chart was given the name of the Hashmi-Asif Covid-19 formula for calculating early common signs and symptoms of Covid-19 for early detection and disease assessment.

**Table 1:** Collected data on common signs and symptoms of Covid-19. All data is shown in percentages.

	**Physical symptoms**		**Mucus membrane**		**Blood cells**			**Liver enzymes**	
	**Fever**	**Cough**	**Dry**	**Hyperemic**	**Leukopenia**	**Lymphopenia**	**Thrombopenia**	**AST**	**ALT**
Guan et al. (28)	43.8	67.8	33.7	~	33.7	87.2	36.2	22.2	21.3
Lu et al. (22)	58.5	48.5		46.2	26.3	3.5	~	14.6	12.3
Holshue et al. (17)	+	+	+	~	+	+	+	+	+
Qiu et al. (23)	36	19	~	~	19	36	~	8.3	5.5
Haung et al. (24)	98	76	~	~	25	76	95	41.3	+
Liu et al. (25)	100	100	~	83.7	66.7	100	~	66.7	16.6
Song et al. (20)	1	–	–	–	–	–	–	–	–
Wang et al. (9)	98.6	59.4	~	~	+	70.3	+	+	+
Jin et al. (21)	84.34	68.91	12.11	~	+	+	+	-	-
Li et al. (6)	+	–	–	–	+	+	_	_	_
Bhatraju et al. (27)	50	88	~	~	4	75	37.5	37.5	28
Gudbjartsson et al. (29)	44.7	30.38	~	13.43	~	~	~	~	~
Zhang et al. (30)	100	100	~	~	-	100	66.33	**↓**100	**↓**100
Bangalore et al. (31)	72	83	~	~	-	100	~	~	~
Goyal et al. (18)	77.1	79.4		~	15.5	90	27	46.5	32
Song et al. (20)	39.2	17.85	~	21.42	~	~	~	~	~
Chow et al. (33)	72	87.5	~	~	~	~	~	~	~
Shi et al. (34)	80.3	34.6	~	2.9	~	+	+	–	–
Wu et al. (35)	26.3	~	~	~	–	100	–	+	+
Richardson et al. (19)	30.7	~	~	~	–	60	~	58.4	39

*+ Present but not calculated, ~ Not reported, –Normal values, ↓ Below normal values*.

### Statistics

We investigated the relationship of frequent appearances of common signs and symptoms with diagnosed Covid-19 cases by Pearson correlation and Spearman correlation coefficient (rho) two-tail (38). Cumulative frequencies of each common sign and symptom were assessed by Chi-square test two tail with Cramer's V strength methods (40). Highly significant symptoms and signs showing correlation were assessed by the linear regression method to establish ostensible correlation. Compiled data was analyzed statistically by using IBM SPSS Version.20.

## Results

Results of 10,172 confirmed Covid-19 cases showed the appearance of signs and symptoms in relation to the pathological progression of Covid-19. Infection leads to initial changes that occurred in blood biomarkers and, when reaching threshold level, produced symptoms (Details are shown in Table 1). All signs and symptoms cumulatively showed a 39.33% sensitivity correlation with the cumulative scoring method and a 48.11% through star values scoring method among all cases evaluated for Covid-19. Data showed that if all the confirmed cases were analyzed before confirmation with the early signs and symptoms at 39.33 and 48.11% with star values, cases could be detected earlier than usual in the course of disease, and would be considered at very high risk of developing Covid-19.

### Statistical Analysis

Twenty studies containing detailed information of 10,172 Covid-19 laboratory confirmed cases showed a common symptomatic correlation with Covid-19 were statistically significant (sig. <0.000) for each sign and symptom. Fever at 43.9% was significant 0.000. Cough at 54.08% and dry mucus membrane at 25.68% values were equally significant 0.000, hyperemic mucus membrane at 17.92% was significant with *p* < 0.005, leukopenia (28.11%) and lymphopenia (64.35%) showed a significance of 0.000. Thrombopenia (35.49%) showed a strong correlation (sig.0.000) with Covid-19 at significant *p* (<0.01). Amino transferases ALT and AST (50.02 and 34.49%, respectively) showed a strong correlation and were statistically significant (<0.001). Thereafter, symptoms holding high sensitivity correlations (star values) with the development of Covid-19 were extracted by linear regression model. Statistical data is shown in Table 2. Symptoms frequency appearance in Covid-19 was assessed by Chi-square method and results shown in Table 2. Fever and lymphopenia frequency showed a similar significance (*P* < 0.000). Cough showed a significance frequent appearance in Covid-19 (*P* > 0.02). Dry mucus membrane and thrombopenia showed a similar significance (*P* < 0.006). Hyperemic mucus membrane did not show a significant value (*P* < 0.062), while aminotransferases showed an equal significance (*P* < 0.001).

**Table 2:** Symptomatic Correlation and frequency with development of Covid-19.

**Symptoms**	**Reported cases Total (10,172)**	**Correlation Sig. (<0.01)**	**Frequency Sig. (<0.05)**
Fever	4466/10172 (43.9%)	<0.000	<0.000
^*^Cough	2398/4434 (54.08%)	<0.000	<0.02
Dry Mucus Membrane	450/1752 (25.68%)	<0.000	<0.006
Hyperemic Mucus	256/1428 (17.92%)	<0.005	<0.062
Leukopenia	498/1771 (28.11%)	<0.000	<0.006
^*^Lymphopenia	5024/7807 (64.35%)	<0.000	<0.000
^*^Thrombopenia	554/1561 (35.49%)	<0.000	<0.006
^*^Elevated ALT	3738/7472 (50.02%)	<0.000	<0.001
Elevated AST	2563/7431 (34.49%)	<0.000	<0.001

(^*^)= Star Values.

### Hashmi-Asif Covid-19 Chart

Symptoms of Covid-19 were classified into early symptoms and late symptoms based on severity. Early symptoms can be a point of consideration for getting early detection. Covid-19 diagnosis could be missed during the early stage because of early symptoms being mild in nature. However, distinct evaluations for Covid-19 could be made by calculating scores of correlated blood biomarkers analysis through the Hashmi-Asif Covid-19 chart as elaborated in Chart 1. Common signs and symptoms were classified according to severity including normal with no disease, milder, moderate, and severe cases. The formula contains a maximum of 39 (15+24) scores, out of which cases with a cumulative scoring ranging from ≥13–22/39 should be considered at high risk to be diagnosed with Covid-19, isolated immediately, and should be evaluated by standard diagnostic procedure RT-PCR for SARS-Cov-2. The formula provides an easy approach to screen the suspects and carriers of Covid-19 3–4 days earlier than current procedures, because oropharyngeal or nasopharyngeal swabs detected positive for SARS-Cov-2 by RT-PCT after an average of 7 days of infection. Blood oxygen saturation does not change much at early stages and the reason was not included in the calculation formula. Oxygen saturation decreases during advanced stages of Covid-19 and time can be saved by taking such early measures. Decreased O_2_ gas in the blood is signifies a critical situation that requires urgent interventions.

**Chart 1:** Hashmi-Asif Covid-19 Assessment Chart.

**Physical Signs and Symptoms**		**Scores**			**Obtained score**
Temperature	≤ 37 Score-1	<37.5 Score-2	37.5–38 Score-3	>38 Score-3	
^*^Cough	Absent Score-1	Productive Score-2	Dry Cough Score-3	Prudent Score-3	
Fatigue	Absent Score-1	From 1 Day Score-2	From 2 days Score-3	>2day Score-3	
Nausea and vomiting	Absent Score-1	Nausea with vomiting Score-2	Vomiting with diarrhea Score-3	Vomiting with abdominal discomfort Score-3	
Mucus membrane	Normal Score-1	Inflamed Score-2	Dry appearance Score-3	Hyperemic Score-3	
				**Total**	
**Blood biomarkers**					
**Leukocytes** 3,800–1,100/μl	5,000–11,000 Score-1	3,800–5,000 >11,000 Score-2	3,500–3,800 Score-3	<3,500 Score-4	
^*^Lymphocytes 1,000–3,900/μl	>2,500 Score-1	1,750–2,500 Score-2	1,000–1,750 Score-3	<1,000 Score-4	
**Neutrophils** 1,900–7,400/μl	1,900–3,500 Score-1	≥3,500 Score-2	1,800–1,900 Score-3	<1,800 Score-4	
^*^Platelets 150,000–400,000/μl	>250 × 10^3^ Score-1	150–250 × 10^3^ Score-2	125–150 × 10^3^ Score-3	<125 × 10^3^ Score-4	
^*^Alanine aminotransferase 10–49U/L	<50 Score-1	50–60 Score-2	60–70 Score-3	>70 Score-4	
**Aspartate Aminotransferase** <33 U/L	<35 Score-1	35–40 Score-2	40–50 Score-3	>50 Score-4	
	Cumulative Scoring ≥13–22/39 should be considered at high risk to be diagnosed with Covid-19 and considered for RT-PCR for SARV-Cov-2.			**Total Score** (Cumulative) No Disease ≤ **12** Mild: **13–22** Moderate: **23–33** Severe: **34–39**	

*Scoring result is directly proportional to Covid-19 status*.

* *Star Values*.

### Scoring at Hashmi-Asif Covid-19 Chart

Scoring on the Hashmi-Asif Covid-19 Chart is based on the course of disease of Covid-19 described in recent reports (17, 18). The course of disease of Covid-19 is divided into four stages by the authors. First, a healthy status scoring 12 on the chart is normal. Second, a milder disease form holding some bodily response in blood bio-markers and slight changes occurring in the values of biomarker. These changed responses include neutrophil based antiviral response, lymphopcytes reduction [because lymphocytes get infected by SARS-Cov-2 (9)], and slight changes in aminotransferases. Milder cases score between 13 and 22 on the Hashmi-Asif covid-19 chart. Moderate disease produced a sufficient response within the body to be measured through blood biomarkers and changes in biomarker values scored 23–33 on the chart. Severe cases showed a full body response to viral attack and scored 34–39.

## Discussion

SARS-Cov-2 is highly contagious, and could spread rigorously throughout the world as it did over mainland China within 40 days, infecting 72,314 people during the Covid-19 China epidemic. The epidemic of Covid-19 in China was attributed to the spreading virus spurred on by asymptomatic characteristics of the disease and late appearances of symptoms due to a long incubation period (6, 37). A longer incubation period means certain opportunities to get prepared and prompt early action against Covid-19 to be opted. Asymptomatic cases may be diagnosed on the onset of the disease and earlier symptoms appearing during the course of the disease also holds a credible opportunity to make an earlier than usual diagnosis. Early detection could only be possible by assessing signs and symptoms evaluated from various studies. Evaluation of collected data provides important clinical features that could provide comprehensive and reliable information for cases suspected of developing Covid-19. Among various studied signs and symptoms, only those which were highly correlated with the development of Covid-19 were considered in this evaluation. Fever of a mild to moderate grade was present in 43.9% cases and cough has the highest correlation with the development of Covid-19, and so appeared in the study. Cough was present in 65% of confirmed cases of Covid-19. However, asymptomatic cases developed without early signs were hard to detect before Covid-19 progressed. Coughing was enormously present during the course of disease and could be either dry or productive in nature and may be accompanied with secondary or former infections or underlying pathologies. A dry cough or dry mucus membrane holds significant correlation with the development of Covid-19. An initial response of SARS-cov-2 by blood biormarkers recorded leukocytosis during the initial stages, followed by an enhanced response from white blood cells to develop severe leukocytosis. These cases of leukocytosis showed more than normal upper limits owing to the presence of an underlying pre-existing pathology or secondary infection, or white blood cells reduced in number to develop leukopenia. Leukopenia and thrombopenia are characteristic features of Covid-19 infection and can be assessed before the onset of symptoms. Lymphopenia developed in severe cases with the increasing pathology of SARS-Cov-2 (8). Elevated AST and ALT are also significantly correlated with Covid-19. The scoring methodology was adopted according to the changing values of signs and symptoms and blood biomarkers in relation to the changing status of disease reported in Covid-19 cases and degree of changes with advancing or reduction in disease severity. Scores on the chart increase with worsening of the severity of the disease and reduces with amelioration. Results are compiled in Table 1. A case report of a Covid-19 patient in the USA described by Holshue et al. describes daily observations of the signs and symptoms of a patient hospitalized for 15 days for Covid-19. The 35-year-old-male presented with a dry cough and fever from 3 days. Laboratory investigations showed Leukopenia and thrombocytopenia along with slightly elevated liver enzymes AST and ALT on day 6 and 7 of the illness, respectively. A physical examination revealed dry mucus membrane while no symptoms of rhinorrhea and pneumonia appeared before the ninth day of illness. Nausea and vomiting appeared on the fourth day of illness (17). Various publications explained concrete aspects of the Covid-19 course of development and presentation. Due to the longer incubation period of SARS Cov-2 (12.5 days), along with other hidden advantages, halting the spreading epidemic would require wise judgement and understanding of pathogenesis. The course of Covid-19 begins with the appearance of early symptoms such as a mild temperature, cough, fatigue, nausea, vomiting, dry mucus membrane or hyperemic, dyspnea, consolidated pneumonia like lungs, accompanied with a decline in blood oxygen saturation, leukopenia, lymphocytopenia, thrombocytopenia, elevation in Aspartate aminotransferase (AST), and Alanine aminotransferase (ALT) (6–8). Goyal et al. reported clinical features of 393 laboratory confirmed cases presenting with fever 77.1%, cough 79.4%, leukopenia 15.5%, lymphopenia 90%, thrombopenia 27%, and elevated AST 46.5% and ALT 32% (18). Richardson et al. reported clinical features of 5,700 Covid-19 confirmed cases presenting with fever 30.7%, lymphopenia 60%, and elevated AST 58.4% and ALT 39% (19). Jin et al. prescribed clinical features analyzed from 74 Covid-19 confirmed cases and found fever and cough with dry mucus membrane 84.34, 68.91, and 12.11%, respectively. Leukopenia, lymphopenia, and thrombopenia were also present but liver enzymes displayed normal values (21). Lu et al. described a detailed analysis of signs and symptoms of covid-19 in children. Lu showed leukopenia was 26.3% in children hospitalized for Covid-19. Lymphocytopenia was 3.5%, increased ALT was 12.3%, and elevated AST was 14.6%. Cases described as asymptomatic were 27/171, symptoms of upper respiratory tract infection were in 33/171, and symptoms of pneumonia were present in 111/171 of hospitalized children (22). Qu et al. prescribed epidemiological features of 36 children diagnosed with Covid-19 in Zheijiang, China. Haiyan observed clinical features of a raised temperature in 36% children, cough in 19%, leukopenia in 19%, and lymphopenia in 36% children diagnosed with Covid-19 on time of admission to hospital. AST was elevated in 8.3% children and ALT was elevated in 5.5% children in the early stage of Covid-19. Pediatric patients are difficult to diagnose and can remain asymptomatic for up to 10 days (23). Huang et al. reported clinical features of hospitalized and laboratory confirmed Covid-19 cases in Wuhan, China. Huang C observed fever in 98% and Cough 76% in Covid-19 cases while blood investigations showed leukopenia 25%, lymphopenia 76%, thrombocytopenia 95%, and elevated AST in 37% (24). Liu et al. reported the detection of 06 children with Covid-19 published in the New England Journal of Medicine and recorded fever (6/6) and cough (6/6) in all children diagnosed with Covid-19 under his study. Pharyngeal congestion was 83.7% (5/6), leukopenia (4/6) 66.7%, lymphopenia recorded in all children (6/6) 100%, and platelets values were at the lower limit <20 × 10^4^ in (3/5) children. Elevated AST was (4/6) 66.3% and elevated ALT was 16.7% (1/6) (25). Wang et al. in another publication that appeared in JAMA described clinical symptoms of fever 98.6%, dry cough 59.4%, and lymphopenia 70.3% in laboratory confirmed Covid-19. They also described leukopenia, thrombocytopenia, and elevated Alanine aminotransferase and aspartate aminotransferase in Covid-19 confirmed cases (26) and Pavan et al. provided clinical features of 24 Covid-19 confirmed cases with fever 50%, cough 88%, leukopenia 04%, lymphopenia 75%, thrombopenia 37.5%, elevated AST 37.5%, and ALT in 28% (27). Guan et al. described symptoms of more severe cases of covid-19 as having fever in 975/1,099 patients who were hospitalized with an average temperature of 38.3°C, having Leukopenia in 33.7% patients hospitalized for Covid-19, lymphocytopenia 87.2%, thrombocytopenia 36.2%, elevated Alanine aminotransferase 21.3% and elevated Aspartate aminotransferase were 22.2%, and physician diagnosed pneumonia 91.1%. Cough was found in 67.8%, fatigue 38.1%, and sputum production in 33.7% of hospitalized patients (28). Gudbjartsson et al. reported fever 44.7%, cough 30.38%, and hyperemic mucus membrane 13.43% in 1,221 laboratory confirmed Covid-19 cases (29). Contrary Young et al. did not show any laboratory findings in one study (32). Zhang et al. prescribed fever, cough, and lymphopenia in all cases and thrombopenia in 66.33% of confirmed cases of Covid-19. He also showed reduced levels in AST and ALT aminotransferases (30). Bangalore et al. reported fever 72%, cough 83%, and lymphopenia in all 18 laboratory confirmed Covid-19 cases (31). Song et al. explained the presence of fever 39.2%, cough 17.85%, and hyperemic mucus membrane 21.42% in 28 patients diagnosed with Covid-19 (32) and Chow et al. reported fever 72% and cough 87.5% in 48 confirmed cases (33). Shi et al. observed fever in 80.3%, cough 34.6%, and hyperemic mucus membrane 2.9% in 416 confirmed cases of Covid-19 (34). Wu et al. reported fever 26.3% and lymphopenia in 38 Covid-19 cases (35). Fen et al. primarily described the epidemiological aspects of Covid-19, and asserted that Covid-19 has a mild course of disease and the mortality rate is 2.3%. According to Fen et al. many mildly infected and some severe cases survived the Covid-19 infection. Symptoms vary from the mild to the severe, the latter of which would demand assisted ventilation. 1.2% of asymptomatic patients were confirmed by laboratory investigation. Many suspects were quarantined from their signs and symptoms (36). Detail features are described in Table 1.

### Significance of Hashmi-Asif Covid-19 Formula

Early detection for Covid-19 in symptomatic and asymptomatic cases showed its ability to isolate Covid-19 cases at an early stage. The calculation chart is provided with double calculation methods to enhance the sensitivity of the outcome. The formula provides an easy approach to screen the suspects and carriers of Covid-19 earlier than previously being diagnosed. The Hashmi-Asif covid-19 formula expedites the ability of health care providers in developing countries lacking appropriate health facilities to diagnose Covid-19. The Hashmi-Asif Covid-19 formula based on the most common early presentations of the Covid-19 and on changing response in signs and symptoms and blood biomarkers has made evaluating Covid-19 easier. By using the formula, Covid-19 can be diagnosed ~72–96 h earlier than it currently can. It will provide ample time to adopt interventions for Covid-19 and to reduce the mortality rate by early management. The chart can be helpful to restrict transmission rates of SARS-Cov-2 ≤ 1, consequently decreasing infection spread in contacts. The Hashmi-Asif covid-19 formula expedites the ability of health care providers in developing countries lacking appropriate facilities to diagnose Covid-19. The Hashmi-Asif Covid-19 formula works via calculation of scores of the most common early presentations of Covid-19. Separate calculations of scoring for signs and symptoms and blood biomarkers made it appropriate for Covid-19 detection and evaluation. The chart also provides a tool to assess whether the status of Covid-19 is either progressing or reducing toward a healthy situation.

## Conclusion

We showed a strong correlation between the early and common signs and symptoms leading to the development of Covid-19 and designed the Hashmi-Asif Covid-19 chart which holds the potential of diagnosing 48.11% of asymptomatic Covid-19 cases earlier than usual. For symptomatic cases of Covid-19, the Hashmi-Asif Covid-19 chart holds a sensitivity of 95% to early detection, which will surely reduce transmission rate and prevent an epidemic outbreak or slow down its spread. The chart is also useful to assess the status of covid-19 in patients through regular scoring. The score decreases with amelioration of the Covid-19 situation. The chart can provide essential information about the efficacy of the management method being applied and whether it is useful or not, whether disease severity is reducing or not, and whether the bodily response is either ameliorating or worsening. This chart will help healthcare workers to implement timely measures for critical patients to save lives by opting for appropriate measures, and to make containment strategies to counter Covid-19.

### Limitations

Our study has various limitations. It is a retrospective study based on reported clinical manifestations and probable courses of disease from available data around the world. Individual data of patients of Covid-19 were less reported and collective analyzed data was evaluated. A prospective study is underway to evaluate the utilization of the Hashmi-Asif Covid-19 assessment chart and its efficacy within domestic Covid-19 patients.

## Data Availability Statement

The datasets generated for this study are available on request to the corresponding author.

## Ethics Statement

Ethical review and approval was not required for the study on human participants in accordance with the local legislation and institutional requirements. Written informed consent for participation was not required for this study in accordance with the national legislation and the institutional requirements.

## Author Contributions

All authors listed have made a substantial, direct and intellectual contribution to the work, and approved it for publication.

## Conflict of Interest

The authors declare that the research was conducted in the absence of any commercial or financial relationships that could be construed as a potential conflict of interest.
